# The Importance of Patient Mindset: Cosmetic Injectable Patient Experience Exploratory Study—Part 1

**DOI:** 10.1093/asjof/ojac043

**Published:** 2022-05-08

**Authors:** Cara B McDonald, Sarah Hart, Steven Liew, Izolda Heydenrych

**Affiliations:** Department of Dermatology, St Vincent's Hospital, Melbourne, Australia

## Abstract

**Background:**

To meet the needs of each individual cosmetic injectable patient, focus is moving toward a detailed, patient-centered, holistic consultation with pretreatment exploration of the patient’s mindset. The Cosmetic Injectables Patient Experience Exploratory Study (CIPEES) was developed to explore patient motivation, mindset, engagement, and factors impacting the patient-practitioner relationship.

**Objectives:**

In order to best meet the needs of individual aesthetic patients, the authors examine the variability and importance of mindset factors in patients seeking cosmetic injectables.

**Methods:**

A study was conducted through an online survey. Participants were asked to respond to a series of statements concerning their thoughts and feelings around appearance, treatment goals, and motivating factors. Participants were asked to select one of the following: “describes me well,” “somewhat describes me,” or “does not describe me.”

**Results:**

In total, 1269 participants completed the relevant survey question. Respondents were 95.6% female and 4.4% male, with ages ranging from 18 to > 65 years old (median 33 years old). Responses were also analyzed according to age group. Data analysis revealed a majority of respondents seeking natural results, with a 15%-20% minority considering a “done” look to be acceptable or even ideal. High numbers of respondents reported being critical of their own appearance and concerned about a specific feature to be “fixed.”

**Conclusions:**

Exploring the nuances of patient mindset will assist practitioners in meeting the unique needs of each patient and may also help them to avoid treating patients whose requirements or expectations are outside their circle of competence.

Cosmetic injectable treatments represent a well-established, rapidly growing segment of aesthetic medicine. While earlier treatment approaches were predominantly technique and result focused, there is currently a growing emphasis on the holistic patient assessment before treatment.^1,2^ Additionally, there is increasing demand for a comprehensive consultation including education on the aging process, facial assessment, upfront discussion about the treatment prioritization, costs, and, not least, the patient’s mindset and motivations. Greater insight into the latter allows practitioners to identify patients with expectations outside of their circle of competence, either from a technical perspective or regarding their emotional needs.

In order to better meet patient’s needs, a “patient-centered approach” considering patient identity, concerns, and preferences is considered beneficial. This method is effective in clarifying patients’ concerns and beliefs, communicating treatment options, increasing empathy, and improving patients’ perception of the provider’s attentiveness.^3^ We propose that gaining a deeper understanding of the cosmetic patient’s psychology should enable aesthetic practitioners to deliver patient-specific pretreatment education for optimizing the intellectual and emotional response to injectable treatment which is vital to patient satisfaction. In everyday cosmetic practice, where objective assessment scales are rarely used, patient satisfaction becomes the primary measure of treatment success. Gaining greater insight into patient emotions, beliefs, and preferences should help set expectations and improve patient satisfaction following injectable treatment, thus leading to better results, decreased complaints, and improved patient retention.

Liew et al published a comprehensive guide to understanding and treating 4 common aesthetic patient archetypes.^4^ Because patient archetypes describe typical examples of certain patient types and their treatment goals, identification of common archetypes can narrow down motivating factors and help the clinician to better meet patient needs. The Beautification Archetype is characterized by patients innately focused on aesthetics, grooming, fashion, and current beauty trends. They are inclined to shop around and are focused on looking good in all situations, particularly on social media. The Transformation Archetype embodies patients desiring a total look, or culturally defined beauty ideal, in order to provide a competitive edge or improve social standing. The Correction Archetype defines the patient who is motivated by a specific congenital or acquired feature negatively impacting their life, such as a traumatic scar or facial palsy. The Positive Aging Archetype is characterized by the patient wishing to minimize visible signs of aging, typically requesting subtle results without changing “who” they are.

While the utilization of the patient archetype broadly allows clinicians to better reach patient-focused aesthetic outcomes, there are innumerable nuances that may be further explored in order to uncover true motivating factors, treatment goals, expectations, and insecurities as well as signs of body dysmorphia. Although seemingly simple, most patients are unable to articulate these feelings adequately, often not being fully aware of themselves.

## METHODS

In order to distinguish patient characteristics and examine their care experience during cosmetic injectable treatments, an online survey was conducted. The Cosmetic Injectables Patient Experience Exploratory Study (CIPEES) was developed to examine patient motivation, mindset, engagement, and salient aspects of the patient-practitioner relationship (Supplemental Material 1). The survey, open to any person who had previously undergone cosmetic injectable treatments, was anonymous and completed online through snowball recruitment. The snowball approach uses a collaborative network to acquire data from a large study population.^5^ The survey was in the English language but open to participants globally. The survey was hosted on SurveyMonkey (San Mateo, CA) and was open for 10 months from September 2020 to June 2021. The study was approved by Human Research Ethics Committee, St Vincent’s Hospital Melbourne (Australia). There were neither incentives nor paid advertisements.

Written consent was provided, by which the participants agreed to the use and analysis of their data. After agreement to proceed, participants were asked to read 13 individual statements concerning their thoughts and feelings around their appearance, and motivation for the cosmetic injectable treatments. The order in which each statement appeared in the survey was randomized. For each statement, participants were asked to select one of the following: “describes me well,” “somewhat describes me,” or “does not describe me.”

The 13 mindset statements in the survey were developed by the authors based on extensive clinical and teaching experience in the field of cosmetic injectable treatments. In combination, the statements were considered to help differentiate and define common patient personas seeking out cosmetic procedures. The statements were tested in a pilot survey of 80 clinic patients to ensure they were easily understood, and a high completion rate was achieved.

## RESULTS

Of the 1430 participants in the CIPEES survey, 95.6% identified as female and 4.4% male, with ages ranging from 18 to > 65 years (median 33 years old). The respondents were made up of residents from 74 countries with 59.0% living in Australia, 10.0% in the United States, 6.2% in the United Kingdom, and small numbers across 71 other countries (Supplemental Material 2). The numbers were insufficient to analyze mindset differences between countries and cultures. The age range and gender of respondents are displayed in Table 1. The question relevant to this paper, exploring patient mindset, was completed by 1269 participants.

**Table 1.** Table Showing Distribution of Age and Gender for Respondents of Cosmetic Injectables Patient Experience Exploratory Study Survey

Sex	Age (y)						Total	
	18-24	25-34	35-44	45-54	55-64	65+	(%)	(n)
Female	8.3%	35.8%	27.5%	19.5%	6.8%	1.8%	95.6%	1367
Male	11.1%	47.6%	17.5%	12.7%	7.9%	1.6%	4.4%	63
Total	8.5%	36.3%	27.1%	19.2%	6.9%	1.8%	100.0%	1430

## DISCUSSION

### Mindset Statements and Considerations

#### Statement 1

You feel that you have a specific feature/area that causes you to be self-conscious and needs/needed to be fixed

Concern regarding a specific feature or area requiring rectification traditionally corresponds with the Correction Archetype, typically characterized by patients with a specific acquired or structural deficit and focused on a discrete treatment request (Table 2). Although this group is said to form a minority of patients seeking cosmetic injectables, the results of this survey show a specific feature of concern to be remarkably common, with 44.6% of respondents feeling that this described them well and 39.7% feeling that this somewhat described them. This equates to a surprisingly high 84% of respondents feeling that they have a specific facial feature warranting attention.

**Table 2.** Statement 1: You Feel That You Have a Specific Feature/Area That Causes You to be Self-Conscious and Needs/Needed to be Fixed

Does not describe me		Somewhat describes me		Describes me well	
15.7%	n = 199	39.7%	n = 504	44.6%	n = 566

The abovementioned result suggests that this mindset is not exclusive to the Correction Archetype, where the practitioner and outsiders would be able to identify the concern, but extends to other archetypes, such as positive aging and beautification. This offers an important insight into patient perception, with the majority focusing on a single concern.

Other studies have found that aging patients are commonly concerned with one area, such as nasolabial folds, without being aware of underlying or causative aging changes such as volume loss elsewhere, even when using a mirror.^6^ Many patients have limited knowledge of either contributory aging factors or negative nonverbal messages communicated by the face. Should they attribute their negative feelings to one specific feature, discussion prior to treatment is needed to set realistic expectations around their presenting concern. 

These findings underline that practitioners should routinely address patients’ tendency to overly focus on a single concern. The healthcare professional (HCP) should use clinical photography to demonstrate the face in its entirety, educate patients about the holistic facial aging process, and set realistic expectations regarding the feature of concern.

The high percentage of patients concerned with a specific feature may contribute to inadvertent unnatural results. “Perception drift” is a term coined by Sabrina Fabi to describe how, after cosmetic treatment, patients may become temporarily more inclined to look at their face “locally” rather than “globally,” potentially leading to fixation on a new flaw after correction of the original concern. As new perceived flaws are perseverated on and addressed, patients continuously develop new baselines and eventually no longer look like themselves.^7^

Although preoccupation with one facial feature appears to be common, clinicians should nevertheless be aware this may also be a symptom of body dysmorphic disorder (BDD).^7,8^ Further patient information should be elucidated to clarify the possibility of BDD, particularly the degree of distress and how their concern is affecting their daily functioning.

It is also important to realize that patients across the gender identification spectrum (LGBTQ+) view aesthetic treatments not as beauty treatments but as a way in which to express how they feel about themselves. There may be treatment requests for a single area, and an attentive history is essential for exploring underlying motivations.^9^

#### Statement 2

You feel that you have aged prematurely and are let down by your appearance

Although most respondents did not feel that they had aged prematurely, approximately 40% did feel that this described them to some degree (Table 3). This group is easy to identify during a consultation, representing a subset of the Positive Aging Archetype. Patients feeling that they have been let down by their appearance tend to think they should look better for their age, therefore seeking restoration of their perceived normal appearance.

**Table 3.** Statement 2: You Feel That You Have Aged Prematurely and Are Let Down by Your Appearance

Does not describe me		Somewhat describes me		Describes me well	
60.4%	n = 767	28.8%	n = 365	10.8%	n = 137

In many cases, these patients have been through stressful life events, grief, chronic illness, or substantial weight fluctuations, feeling that these hardships have influenced their appearance. They are not necessarily seeking to remove signs of aging, seeking instead to look how they feel they should. This group tends to be extremely satisfied with the subtle treatment and a reduction in the nonverbal, negative messages that they communicate. This is a satisfying group to deal with from an HCP’s perspective.

#### Statement 3

You are very proactive and want to reduce all signs of aging 

This group, which may include a subset of the Positive Aging Archetype, is more proactive than those described above (statement 2) and generally doesn’t want to look at their age (Table 4). Most respondents agreed at least to some degree with this statement, with similar results across all generations. Although this should not be surprising in a group who are already consumers of cosmetic injectables, it is interesting to appreciate that there is considerable fear of the aging process and associated signs. This highlights the need to communicate clearly that it is not usually possible to remove “all signs” of aging without destroying unique features and that, in most cases, this is not the desired outcome for either the patient or practitioner.

**Table 4.** Statement 3: You Are Very Proactive and Want to Reduce all Signs of Aging

Does not describe me		Somewhat describes me		Describes me well	
11.6%	n = 147	40.1%	n = 509	48.3%	n = 613

#### Statement 4

You want to slow down or reduce the signs of aging but want to maintain a natural look

Overall, only 1.7% of respondents disagreed with this statement, selecting that it did not describe them (Table 5). Although the results may be skewed by geographical and cultural selection bias, this survey does suggest that most consumers seeking injectable treatments have the desire to maintain at least a somewhat natural aesthetic. However, “natural” is a subjective term that may be interpreted differently between various generations and social circles.

**Table 5.** Statement 4: You Want to Slow Down or Reduce the Signs of Aging but Want to Maintain a Natural Look

Does not describe me		Somewhat describes me		Describes me well	
1.7%	n = 22	12.3%	n = 156	86.0%	n = 1091

These results reflect a different reality to that seemingly portrayed by society, which may be partially explained by the unknown number of concomitant members of the population with imperceptible cosmetic treatments. However, it is probable that there are a substantial number of cosmetic patients displaying conspicuous results despite their apparent desire to maintain a natural look. Causes for this may include, but are not limited to, a distorted idea of “natural,” inappropriate yet fulfilled treatment requests, under-skilled injectors, upselling of product, and/or insufficient time dedicated to comprehensive consultation.

It is not possible to discern whether the 14% of patients who felt this did not describe them well were less concerned about reducing signs of aging or did not want to retain a natural look. Subsequent statements may provide more clarity.

#### Statement 5

You love the cosmetically enhanced and “done” look

This question explicitly investigates the desire for a “done” look, revealing overall a small group of patients (5.7%) actively seeking an unnatural result (Table 6). This increased to 12.6% in the youngest age group (18-24 years) and dropped down to 2.6% in the older respondents (>55 years). A further 17.9% of respondents overall felt it described them somewhat. This group may include Beautification and Transformation Archetypes. This minority group showing conspicuous signs of cosmetic treatment may create a strong impression among injectable-naïve consumers that all cosmetic injectable procedures will be apparent to outsiders. In contrast, a 76.4% majority rejected the “done” aesthetic outright, indicating that it did not describe them at all. In the authors’ clinical experience, fear of an unnatural-looking result is a major deterrent to treatment for this group of patients.

**Table 6.** Statement 5: You Love the Cosmetically Enhanced and “Done” Look

Does not describe me		Somewhat describes me		Describes me well	
76.4%	n = 970	17.9%	n = 227	5.7%	n = 72

This question reveals there are 2 dichotomously opposed patient groups—a minority group seeking a “done” look and a majority group who consider such a result to be a completely unacceptable outcome. For the practitioner, each of these groups requires different consultation skills, education, injectable techniques, and marketing. Ideally, practitioners should be aware of their own innate aesthetic preference for either “natural” or “overdone” and consider their possible inability to meet patient expectations with alternative aesthetic ideals.

#### Statement 6

You place a high value on your appearance and are aiming for “next level” beautification

Although the results of statement 4 reveal that most respondents aspire to natural results, it is apparent that most of the respondents also place a high value on appearance and are often seeking “next level” beautification (Table 7). Approximately two-thirds of respondents at least somewhat agreed with this statement. The age of respondents who felt this described them well (20.6%) showed a similar distribution to the overall group.

**Table 7.** Statement 6: You Place a High Value on Your Appearance and Are Aiming for “Next Level” Beautification

Does not describe me		Somewhat describes me		Describes me well	
35.8%	n = 454	43.7%	n = 554	20.6%	n = 261

Considering statements 4 and 6 simultaneously, there appears to be a mismatch in expectations of consumers who aspire to “next level” beautification, while also wishing to remain within the spectrum of a “natural” aesthetic. There is a fine line between “next level” beautification, or a striking appearance, and an overdone aesthetic with the loss of unique facial features. Many practitioners do not have the aesthetic eye or technical skillset to walk this line, and consequently, consumers seeking beautification inadvertently receive excessive augmentation, thereby gaining an unnatural appearance. Critically, patients seeking “next level” beautification should be probed around their preference for a natural aesthetic before undergoing beautification treatments and educated thoroughly about the possible outcomes.

#### Statement 7

You are critical of your own appearance and either avoid looking in the mirror or obsess over what is “wrong”

It is of substantial concern that such a high proportion of respondents are overly critical of their own appearance. Although self-criticism is inherent to human nature, this question sought out excessively critical respondents who either avoided their own reflection or obsessed over what they felt is wrong, which are classic features of BDD (Table 8).^10^ This high incidence approaches the rates of BDD among plastic surgery patients, which is cited as 2.2%to 56.7%,^11^ in comparison to usual community rates of 0.7% to 3%.^8^

**Table 8.** Statement 7: You Are Critical of Your Own Appearance and Either Avoid Looking in the Mirror or Obsess Over What Is “Wrong”

Does not describe me		Somewhat describes me		Describes me well	
38.5%	n = 489	35.4%	n = 449	26.1%	n = 331

However, the respondent’s definition of being self-critical and obsessing over their appearance may well differ from the levels required to meet the diagnostic criteria for BDD.^12^ Further questioning is required to elucidate whether the patient is describing true BDD or more “normal” appearance-related anxiety which does not affect daily functioning. Appearance dissatisfaction may be higher in those seeking cosmetic injectable treatments or could simply reflect trends seen in the general population. A 1997 multinational body image survey of 4000 participants found 56% of females to be dissatisfied with their appearance.^13^ This appearance-related concern has increased drastically with the advent of social media.^14^

For diagnosis of BDD, Diagnostic and Statistical Manual of Mental Disorders 5 (DSM-5) requires spending excessive time (on average, 3-8 hours a day) thinking about body areas viewed as unattractive or abnormal, to the point where these concerns cause clinically significant distress or impairment in functioning.^12^ It is important to understand that there is a continuum from self-improvement to self-loathing, and all HCPs who deliver cosmetic injectables should familiarize themselves with the diagnostic criteria of BDD, be alert to the possibility of this disorder, and be ready to refer their patients for psychological help when indicated.

Importantly, a recent study has highlighted the fact that one-third of participants on Zoom calls develop appearance dissatisfaction around new facial or body areas, and that this is apparent not only in those with underlying BDD but also in normal individuals.^15^

#### Statement 8

You will forego other things in order to undergo more cosmetic treatments

A relatively small proportion of respondents felt that they would forego other things in order to undergo cosmetic treatment, with 14.3% agreeing that this described them well (Table 9). This was remarkably consistent across age groups despite being a hallmark feature of the Beautification Archetype.^4^ This behavior should be carefully explored by the treating practitioner, because while it has been found that cosmetic treatments can improve psychological and social functioning,^16^ expectations may exceed the likely improvement with the unfounded belief that cosmetic treatments will lead to additional benefits such as a new partner, a better job, or an improved social status. This can lead to intense patient dissatisfaction when not manifesting.

**Table 9.** Statement 8: You Will Forego Other Things in Order to Undergo More Cosmetic Treatments

Does not describe me		Somewhat describes me		Describes me well	
50.0%	n = 634	35.8%	n = 454	14.3%	n = 181

#### Statement 9

You are happy to invest time and money in the best quality treatments and products

The respondents of this survey were mostly looking for quality in their aesthetic treatments, suggesting that consumers in this survey were willing to pay for quality service and products as opposed to seeking low-cost alternatives and discounts (Table 10). Although certain practitioners and clinics will specifically cater to those seeking a lower price point, the results suggest that the majority would be happy to pay more if they know they are receiving better quality of care, service, and products. Surprisingly, the younger generations also felt that they were more likely to invest in the best quality treatments with 69.9% of those aged 18 to 24 years saying that this describes them well.

**Table 10.** Statement 9: You Are Happy to Invest Time and Money in the Best Quality Treatments and Products

Does not describe me		Somewhat describes me		Describes me well	
2.1%	n = 26	33.3%	n = 423	64.6%	n = 820

#### Statement 10

You don’t want to seem vain or look “done” but want to look more like “yourself” again

Among certain populations, there is still a stigma attached to undergoing cosmetic procedures due to the perception that those seeking aesthetic procedures are excessively vain, have problematic self-esteem issues, or possibly even BDD (Table 11). While many individuals merely appreciate their postprocedural appearance, it is important for HCPs to recognize the high frequency of background anxiety pertaining to perceived vanity.

**Table 11.** Statement 10: You Don’t Want to Seem Vain or Look “Done” but Want to Look More Like “Yourself” Again

Does not describe me		Somewhat describes me		Describes me well	
13.8%	n = 175	29.7%	n = 377	56.5%	n = 717

Clear differences were observed across age groups. Approximately, 73.3% of those aged 45 to 54 years and 69.8% of those >55 years felt that this statement described them well, suggesting high levels of concern in these age groups that seeking cosmetic injectable treatments may make them appear to be vain. This contrasted with 52.3% of those aged 25 to 44 years and only 31.1% of those <24 years.

Given that most respondents agreed at least to some degree with the statement, with only 13.8% feeling this did not describe them, it is helpful for HCPs to preempt this possible concern and alleviate anxiety before treatment. Equally, it is useful to note that yet again a minority (13.8%) of patients are overtly unopposed to looking “done.”

#### Statement 11

You choose your cosmetic injectable treatments by the best price/deal available

These results confirm those seen from statement 9 in that most respondents were not primarily seeking out low price or treatments (Table 12). However, 26.6% of respondents did somewhat agree that they would choose injectable treatments based on the best price deal and 8.4% agreed completely with this. Again, this reflects a spread across all age groups. These results suggest that there is an important market for discount cosmetic injectable treatments, although they did not appeal to most respondents in this survey.

**Table 12.** Statement 11: You Choose Your Cosmetic Injectable Treatments by the Best Price/Deal Available

Does not describe me		Somewhat describes me		Describes me well	
65.1%	n = 826	26.6%	n = 337	8.4%	n = 106

#### Statement 12

You worry about looking “done” and don’t want others to notice you have had treatment

Once again, a 17.7% minority of patients embrace a more unnatural, enhanced appearance, being unconcerned about looking “done,” while a majority wish to avoid noticeable tell-tale signs of cosmetic treatments (Table 13). The younger age groups were slightly less concerned about looking “done,” with 28.2% of those <24 years saying this did not describe them.

**Table 13.** Statement 12: You Worry About Looking “Done” and Don’t Want Others to Notice You Have Had Treatment

Does not describe me		Somewhat describes me		Describes me well	
17.6%	n = 223	30.9%	n = 392	51.5%	n = 654

Individuals with overt previous cosmetic procedures, or those openly sharing and publicizing their procedures, may lead cosmetic-naïve consumers to believe that all cosmetic treatments will be obviously discernible to the outsider. It is of great importance for individual HCPs and the industry to educate those interested in undergoing treatment about the possibility and preference for natural and undetectable cosmetic injectables results.

#### Statement 13

Even after treatment, you don’t feel happy with your appearance

The results suggest that most respondents (64.5%) feel happy with their appearance after undergoing cosmetic injectables treatments (Table 14). Despite a noteworthy proportion feeling only somewhat happy (28.7%), there was only a small percentage agreeing that they don’t feel happy at all (6.9%). Interestingly, there was little variation between age groups although it was slightly higher amongst the youngest group (<25 years) at 11.6%.

**Table 14.** Statement 13: Even After Treatment, You Don’t Feel Happy With Your Appearance

Does not describe me		Somewhat describes me		Describes me well	
64.5%	n = 818	28.7%	n = 364	6.9%	n = 87

In the author’s opinion, there are several potential reasons for postprocedural dissatisfaction, including a truly suboptimal cosmetic outcome due to inappropriate patient selection or treatment choice, and underlying low self-esteem or BDD. Another reason is the inability of the HCP to set realistic expectations before treatment. Technical outcomes may be affected by many variables, including underlying patient factors, treatment selection, and the skill set of the practitioner, but, in many cases, the expected outcome and ability to reach treatment goals have not been fully explained to the patient before the procedure.

### The Consultation

Assessment of the patient’s mindset starts as early as their first interaction with receptionists and booking managers. Their behavior toward support staff may reveal valuable information about a patient’s state of mind and should be relayed to the practitioner. A comprehensive consultation poses the next opportunity to gather information.

The aesthetic consultation must cover a comprehensive medical, cosmetic, social, and psychological history, and include a full-face assessment, baseline photography, and education around the anatomical aging process. The consultation process should seek out “red flags” or inappropriate candidates and explore contraindications such as pregnancy, allergies, and certain underlying medical conditions. The patient and practitioner need to review treatment options and, using a shared decision-making process, determine the treatment to be performed.^17,18^ Thereafter, fully informed consent for treatment, management of possible complications, and financial aspects is mandatory. The time allocated for an aesthetic consultation is often insufficient, resulting in a less comprehensive exploration of the patient’s mindset and emotional needs than is required to truly understand the patient.

The nuances and variability in patient mindset seen in these data emphasize the value of empathic communication and building rapport with the patient before treatment. In order to meet a patient’s needs, the practitioner should build connections and gently probe their innermost thoughts by giving priority to time and listening. Valuing the time and patient mindset during the consultation will enable summarizing back and accurately reflecting the patient’s needs, wants, aspirations, and insecurities, thereby building rapport and a long-term trusting relationship.

### Natural vs Done

Analysis of the data demonstrates 2 distinct, dichotomous groups: a majority group seeking natural results, and a 15% to 20% minority considering a conspicuous look to be acceptable or even ideal. This is somewhat influenced by generation, but age alone does not appear to define individual preferences. This knowledge enables the practitioner to adjust their consultation and education process according to the patient group, thereby educating the natural patient that subtle results are possible and helping the overdone patient to have more realistic perceptions and expectations.

The approach to each group differs regarding communication skills, patient education, treatment plans, injection techniques, and even marketing. It may be difficult to expertly service both patient groups in the same practice, and practitioners may find it most rewarding to elect which patient group matches their own innate aesthetic preference and to adjust their approach accordingly. Once again, the distinct needs of the growing cohort of gender-diverse patients need to be borne in mind.

### Self Perception and Self Image

In this survey, reasonably high rates of some red flag symptoms associated with BDD were identified. These included the feeling that a certain feature needed to be “fixed,” obsessing over what is “wrong” and ongoing dissatisfaction following treatment. However, essential BDD diagnostic criteria such as degree of distress and interruption of daily life were not investigated, so further research would be required to clarify the incidence of BDD in this population. Aesthetic practitioners should be familiar with the diagnostic criteria of BDD and, when appropriate, be aligned with and refer to a psychologist.

The survey also revealed an 84% majority of patients tending to identify a single facial feature as requiring treatment. This limited perspective can lead to an excessive focus on correcting one area, thus leading to overtreatment and an unnatural appearance. Practitioners should guide patients to see their faces holistically, using photographs during the consultation, in order to address this common tendency.^6^

By exploring the thoughts and feelings of patients undergoing cosmetic treatments, we can more easily predict how they may react after treatment and prepare them for their likely emotional experiences. Negativity bias or the preferential weighting of negative thoughts, emotions, and features over and above positive ones should be addressed during consultation rather than postprocedure. While an innate part of human nature, it may be overpowering in highly self-critical or high achieving individuals. Educating relevant patients on negativity bias before treatment will help them understand that they may naturally continue to focus on what they don’t like, or what still “needs” to be done, despite significant improvement.

The authors acknowledge the limitations of this study, particularly with regard to the inherent selection and response bias with an online survey format, and the limitations of using a relatively small number of predetermined mindset statements. Further detailed qualitative research would be required to understand how aesthetic consultation impacts patient outcomes. The mindset statements chosen for this survey are far from all-inclusive with regard to motivating factors, pre-conceived ideas, self-evaluation, and treatment goals but represent several common thoughts and feelings expressed during the authors’ experience in cosmetic consultations.

Each individual practitioner will have a unique circle of competence, the area of practice in which they can successfully meet the technical and emotional needs of the patient. Practitioners will benefit from prioritizing a thorough, holistic consultation with a focus on mindset in order to meet the needs of the patient and avoid treating those outside their circle of competence.

## CONCLUSIONS

The CIPEES survey results give practitioners greater insight into patient thoughts and feelings beyond the cosmetic patient archetypes. A greater understanding of the patient’s preexisting perceptions, expectations, insecurities, goals, and motivations can help the practitioner to set realistic expectations before treatment, which is the cornerstone of patient satisfaction. An excellent outcome in the patient’s mind is the one that meets their expectations.

Polish American scientist and philosopher Alfred Korzybski famously remarked that “the map is not the territory.” Similarly, the patient is by no means the archetype. The authors recommend that practitioners prioritize time to fully investigate their patients’ unique mindsets, rather than making generalized assumptions regarding their needs or wants. Practitioners will benefit from understanding the nuances of patient mindset to improve patient satisfaction and avoid treating patients whose needs or expectations fall beyond their circle of competence. 

## Supplementary Material

ojac043_suppl_Supplementary_Material_S1

ojac043_suppl_Supplementary_Material_S2
